# Anatomical Versus Non-Anatomical Pulmonary Metastasectomy: European Multicentre Analysis

**DOI:** 10.3390/cancers18061037

**Published:** 2026-03-23

**Authors:** Elena Prisciandaro, Luca Bertolaccini, Steffen Fieuws, Andrea Cara, Lorenzo Spaggiari, Lin Huang, René H. Petersen, Marco Lucchi, Maria G. Mastromarino, Annalisa Barbarossa, Paul De Leyn, Matteo Roffinella, Enrico Ruffini, Abid Donlagic, Michel Gonzalez, Marta G. Fuentes-Gago, Clara Forcada-Barreda, Maria T. Congedo, Stefano Margaritora, Yaniss Belaroussi, Matthieu Thumerel, Jérémy Tricard, Pierre Felix, Nina Lebeda, Isabelle Opitz, Angela De Palma, Giuseppe Marulli, Cesare Braggio, Pascal A. Thomas, Frankie Mbadinga, Jean-Marc Baste, Bihter Sayan, Bedrettin Yildizeli, Jeroen Dekervel, Dirk E. Van Raemdonck, Walter Weder, Laurens J. Ceulemans

**Affiliations:** 1 Department of Thoracic Surgery, University Hospitals of Brussels, 1070 Brussels, Belgium; 2 Department of Medical and Surgical Sciences (DIMEC), University of Bologna, 40126 Bologna, Italy; 3 Department of Thoracic Surgery, IEO, European Institute of Oncology IRCCS, 20141 Milano, Italy; luca.bertolaccini@unimi.it (L.B.); cara.andrea.md@gmail.com (A.C.); lorenzo.spaggiari@ieo.it (L.S.); 4 Department of Oncology and Hemato-Oncology, University of Milan, 20122 Milano, Italy; 5 KU Leuven, Interuniversity Institute for Biostatistics and Statistical Bioinformatics, 3000 Leuven, Belgium; steffen.fieuws@kuleuven.be; 6 Department of Cardiothoracic Surgery, Copenhagen University Hospital, Rigshospitalet, 2100 Copenhagen, Denmark; lin.huang@regionh.dk (L.H.); rene.horseben.petersen@regionh.dk (R.H.P.); 7 Division of Thoracic Surgery, Cardiac, Thoracic and Vascular Department, University Hospital of Pisa, 56124 Pisa, Italy; marco.lucchi@unipi.it (M.L.); mgmastromarino@gmail.com (M.G.M.); 8 Department of Thoracic Surgery, University Hospitals Leuven, 3000 Leuven, Belgium; annalisa.barbarossa@kuleuven.be (A.B.); paul.deleyn@uzleuven.be (P.D.L.); dirk.vanraemdonck@uzleuven.be (D.E.V.R.); laurens.ceulemans@uzleuven.be (L.J.C.); 9 KU Leuven, Laboratory of Respiratory Diseases and Thoracic Surgery (BREATHE), CHROMETA, 3000 Leuven, Belgium; 10 Department of Thoracic Surgery, Azienda Ospedaliera Universitaria Città della Salute e della Scienza di Torino, 10126 Torino, Italy; matteo.roffinella@gmail.com; 11 Department of Surgical Science, Section of Thoracic Surgery, University of Torino, 10126 Torino, Italy; enrico.ruffini@unito.it; 12 Service of Thoracic Surgery, University Hospital of Lausanne, 1011 Lausanne, Switzerland; abid.donlagic@unil.ch (A.D.); michel.gonzalez@chuv.ch (M.G.); 13 Department of Thoracic Surgery, Salamanca University Hospital, 37007 Salamanca, Spain; martagfg@usal.es (M.G.F.-G.); clarafb4@gmail.com (C.F.-B.); 14 Unit of Thoracic Surgery, Fondazione Policlinico Universitario A. Gemelli IRCCS, 00168 Roma, Italy; mariateresa.congedo@policlinicogemelli.it (M.T.C.); stefano.margaritora@unicatt.it (S.M.); 15 Department of Thoracic Surgery, University Hospital Bordeaux, 33604 Pessac, France; yaniss.belaroussi@chu-bordeaux.fr (Y.B.); matthieu.thumerel@chu-bordeaux.fr (M.T.); 16 Department of Cardiac and Thoracic Surgery, University Hospital Limoges, 87042 Limoges, France; jeremy.tricard@etu.unllim.fr (J.T.); felix-pierre@hotmail.fr (P.F.); 17 Department of Thoracic Surgery, University Hospital Zürich, 8091 Zürich, Switzerland; lebedanina@gmail.com (N.L.); isabelle.schmitt-opitz@usz.ch (I.O.); 18 Section of Thoracic Surgery, Department of Precision and Regenerative Medicine and Ionian Area, University of Bari “Aldo Moro”, 70124 Bari, Italy; angela.depalma@uniba.it (A.D.P.); giuseppe.marulli@hunimed.eu (G.M.); 19 Department of Thoracic Surgery, Lung Transplantation and Oesophageal Diseases, North Hospital, 13015 Marseille, France; cesare.braggio@ap-hm.fr (C.B.); pascalalexandre.thomas@ap-hm.hr (P.A.T.); 20 Department of General and Cardiothoracic Surgery, University Hospital Rouen, 76031 Rouen, France; frankie.mbadinga@chu-rouen.fr (F.M.); jean-marc.baste@chu-rouen.fr (J.-M.B.); 21 Department of Thoracic Surgery, Marmara University School of Medicine, 34854 Istanbul, Turkey; bihter_sayan@windowslive.com (B.S.); byildizeli@marmara.edu.tr (B.Y.); 22 Department of Digestive Oncology, University Hospitals Leuven, 3000 Leuven, Belgium; jeroen.dekervel@uzleuven.be; 23 Department of Thoracic Surgery, Bethanien Klinik, 8044 Zürich, Switzerland; walter.weder@hin.ch

**Keywords:** pulmonary metastases, pulmonary metastasectomy, lung metastases, lung metastasectomy, overall survival, recurrence-free survival

## Abstract

**Objectives**: Pulmonary metastasectomy is not a standardised procedure, with no consensus regarding the optimal extent of lung resection. This international multicentre study aimed at comparing short and long-term outcomes of anatomical versus non-anatomical pulmonary metastasectomy. **Methods**: Retrospective database including 1647 patients aged ≥18 years, who underwent curative intent pulmonary metastasectomy between January 2010 and December 2018 at 15 European centres. Patients who underwent pneumonectomy, previous metastasectomies, and/or suffered from extrapulmonary recurrence at the time of lung surgery were excluded. Primary endpoint was overall survival. Secondary endpoints were recurrence-free survival and 30-day morbidity. Differences between the two groups were analysed using 3:1 matching. **Results**: In the matched cohort, 324 patients underwent anatomical resection, and 830 patients underwent non-anatomical resection. Five-year overall survival was 62.0%. Averaged over the entire follow-up, there was no significant difference in overall survival between the two groups (HR = 1.122, 95% CI = 0.909–1.385, *p* = 0.283). In the early period following pulmonary metastasectomy, anatomical resections were associated with worse overall survival (HR = 1.549, 95% CI = 1.135–2.114, *p* = 0.006). The difference in any-site recurrence-free survival between the two groups was not significant (HR = 0.832, 95% CI = 0.690–1.002, *p* = 0.053). Locoregional recurrence-free survival was significantly longer after anatomical resection (HR = 0.651, 95% CI = 0.520–0.817, *p* < 0.001). Thirty-day morbidity was significantly higher after anatomical resection (22.2% versus 13.7% for non-anatomical resections, *p* = 0.001). **Conclusions**: In a highly selected cohort, non-anatomical resection showed comparable survival and lower morbidity compared to anatomical resections, supporting the surgical strategy of favouring limited resections whenever technically and oncologically feasible. Anatomical resections remain a valid option in selected cases with acceptable outcomes.

## Simple Summary

Pulmonary metastasectomy is a commonly performed surgical procedure, but there is still no agreement on how much lung tissue should be removed to achieve the best outcomes. Some surgeons prefer anatomical resections, while others favour more limited, non-anatomical resections. In this international multicentre study, we compared short- and long-term outcomes of these two surgical approaches in patients undergoing surgery with curative intent for lung metastases. By analysing data from a large European cohort, we aimed to clarify whether one approach offers advantages in terms of survival, disease recurrence, and postoperative complications. Our findings show that non-anatomical resections provide survival outcomes comparable to anatomical resections, with a lower risk of postoperative complications, while anatomical resections may reduce the risk of local recurrence in selected cases. These results support a tailored surgical approach, helping surgeons balance oncological effectiveness with preservation of lung function.

## 1. Introduction

Pulmonary metastasectomy (PM) has become a routine practice worldwide, accounting for approximately 9% of all lung resections performed in Europe [1]. Indications for PM have broadened over the last decades [2,3] owing to significant improvements in imaging modalities, surgical techniques, and oncological therapies. As a result, surgical resection of lung metastases with a curative intent is now included in the multidisciplinary approach of selected patients with stage IV disease [4,5,6].

However, due to lack of consensus, appropriate eligibility criteria, surgical techniques, and treatment options have not been adequately addressed by current recommendations [7]. Limited tumour burden (controlled primary tumour, no extrapulmonary disease), low surgical risk, and the possibility to achieve radical (R0) resection are considered essential prerequisites for PM [5]. In this subset of carefully selected patients, PM may attain considerable survival benefits with acceptable morbidity [4,7].

Curative-intent surgery should ensure completeness of resection while preserving respiratory function, to minimise complications and allow potential subsequent PMs. In this regard, non-anatomical resections (nARs) (wedge resections, precision tumourectomies) are generally preferred [3]. Anatomical resections (ARs) (segmentectomies, lobectomies) have a greater impact on cardiopulmonary reserve and are therefore less frequently performed. However, due to the location and size of the lesions, AR may sometimes be the only surgical option. Pneumonectomies are rarely indicated, due to high morbidity and mortality [4,7,8,9,10].

On the other hand, nARs are associated with more narrow resection margins, a risk factor for recurrence and worse survival after PM [11], while ARs are considered more radical and oncologically safer. A systematic review from our group assessed survival differences between ARs and nARs for PM; however, it only included three studies on lung metastases from colorectal cancer, and a meta-analysis was not feasible [12].

The growing interest in the oncological outcomes of limited pulmonary resections is shown by recently published randomised trials, demonstrating the non-inferiority of sublobar resections to lobectomy in terms of survival, for patients with small-sized, peripheral, node-negative primary lung cancer [13,14]. Although these trials were conducted in the setting of primary lung cancer and their findings cannot be directly extrapolated to pulmonary metastases, they have contributed to a broader discussion regarding the oncological adequacy of parenchyma-sparing surgical strategies.

In contrast, the body of knowledge on PM mainly consists of retrospective series [5], and current surgical approaches are evaluated on a case-by-case basis [4].

To support surgical decision-making, we designed an international multicentre research project with the aim of investigating the impact of the extent of resection on short- and long-term outcomes in a large cohort of patients who underwent PM.

## 2. Materials and Methods

The results of the present study are reported in accordance with the Strengthening the Reporting of Observational Studies in Epidemiology (STROBE) guidelines. A STROBE checklist is provided in Appendix A (Table A1) [15].

A retrospective multicentre analysis was performed on a prospectively collected database of patients who underwent PM from January 2010 to December 2018 at 15 European Centres, listed in Appendix B. Institutional Ethics Committees approved the study (IEO 1438, 13 January 2021) in agreement with the General Data Protection Regulation. Due to the retrospective nature of the study, written patient informed consent was waived. Relevant data were retrieved from medical records and collected in a purpose-built Research Electronic Data Capture (REDCap) database [16,17]. When possible, information on events after discharge was collected from medical records, e-mails and/or phone interviews. Follow-up was closed in December 2022. Eligibility criteria were (1) patients aged 18 or older, (2) who underwent PM as their first metastasectomy, (3) PM performed with curative intent (macroscopic complete resection of all lung metastases), (4) PM performed for extrathoracic solid tumour metastases (but including oesophageal tumours), (5) clinical, radiological and/or histological evidence of locoregional control of the primary malignancy at the moment of PM. Exclusion criteria were (1) pneumonectomies, (2) patients who underwent PM prior to the considered period (2010–2018), (3) patients who underwent a metastasectomy of other anatomical sites prior to PM, (4) PM performed for non-solid tumours (haematologic malignancies), (5) performed with diagnostic intent, (6) clinical, radiological and/or histological evidence of extrapulmonary recurrence at the time of lung surgery.

The primary outcome was overall survival (OS). OS was defined as the time interval between PM and death or the last follow-up visit/interview. Patients with no information on OS were removed from the analyses.

Recurrence-free survival (RFS) was defined as the time interval between PM and detection of recurrence or death. Patients alive without any recurrence were censored at last follow-up. Patients without information on recurrence were removed from the RFS analyses. Any-site recurrence was defined as tumour relapse occurring at any site after PM. Locoregional recurrence was defined as tumour relapse occurring in the lung, pleura, hilar and/or mediastinal lymph node, and trachea.

Disease-free interval (DFI) was defined as the time interval between primary tumour diagnosis and diagnosis of lung metastases (or, if unknown, date of PM).

A time interval between primary tumour diagnosis and PM < 6 months corresponded to synchronous disease, while an interval ≥ 6 months corresponded to metachronous disease.

The sites of the primary malignancy were categorised as follows: (a) head and neck: ear, nose, tongue, pharynx, larynx, salivary glands/parotid, thyroid, and other, (b) digestive system: oesophagus, stomach, small bowel, colon and rectum, liver, gallbladder and biliary ducts, pancreas, and other, (c) urogenital/male reproductive system: kidney and ureters, urinary bladder and urethra, prostate and seminal vesicles, testicle, and other, (d) breasts/female reproductive system: breasts, uterus and adnexa, ovary, vagina, and other, (e) skin, (f) nervous system, (g) bone, muscle, vessel and soft tissues, (h) others.

The histology of the primary tumour was categorised as: adenocarcinoma, squamous cell carcinoma, sarcoma, germ cell tumour, melanoma, and others.

The American Society of Anesthesiologists (ASA) Physical Status Classification System [18] was used for perioperative patient assessment.

The Clavien-Dindo classification [19] was followed for grading postoperative complications occurring within 30 days after PM.

Quantitative variables were expressed as mean with standard deviation (SD) or median with interquartile range (IQR) 25–75%, whereas frequencies and percentages were given for nominal variables. Kaplan–Meier estimates and 95% confidence intervals (CI) were reported for OS and RFS. Using Cox regression, two approaches were used to assess differences in OS, any-site RFS, and locoregional RFS between AR and nAR patients. First, a multivariable Cox model was fitted on the total dataset, correcting for the following confounders: age at PM (continuous), sex (male versus female), number of confirmed metastases (1, 2–4, >4), size of the largest confirmed metastasis (<1 cm, 1–3 cm, >3 cm) and induction therapy before PM (yes versus no). The variables defined as confounders were selected based on previously published papers and on the opinions of three experienced authors (EP, LB, and LJC). ‘Centre’ was added as a random effect to handle the potential correlation between patients of the same centre. Second, a univariable Cox model was fitted on a dataset obtained after performing a 3:1 matching on a propensity score (three nAR patients for one AR patient). The matching was based on a propensity score derived from a multivariable logistic regression, using a calliper of 0.30 (on the logit scale). The variables defined as confounders in the multivariable model were used as predictors in the propensity model. However, an exact match was required for the categorical variables, hence differences in the propensity score between the AR patient and the nAR patients were completely determined by the differences in age. The matched dataset was obtained using a greedy matching algorithm. In the Cox models, robust standard errors [20] were used to take the matching into account. Note that AR patients for whom only one or two nAR patients could be found were also included in the analysis. Therefore, the results presented were obtained after weighting, i.e., a nAR patient in a cluster with less than 3 nAR patients received more weight than a nAR patient in a cluster with three nAR patients. Analyses in the matched sample were stratified on centre. In the Cox models, the proportional hazard assumption was verified with a test of Lin, Wei, and Ying [21]. Results were presented from an analysis using restricted cubic splines allowing the effect of AR to vary over time in a flexible way [22] when the proportional hazard assumption was violated. To further illustrate the time-varying effect of AR, a segmented model was fitted, allowing the effect of AR to differ before and after a cutpoint. The cutpoint was determined as the point maximising the likelihood, and a 95% bootstrap percentile confidence interval was reported.

The results of this model need to be interpreted with caution, since they do not reflect the uncertainty in the estimation of the cut-point.

Subgroup analyses were performed as a function of primary tumour site and histology. All analyses were performed using SAS software, version 9.4 of the SAS System for Windows [23].

## 3. Results

### 3.1. Unmatched Cohort Analyses

From 15 European centres, a total of 1867 subjects who underwent PM were included. After excluding 220 subjects (reasons for exclusion are detailed in Appendix C, Table A2), 1647 patients were analysed. Median age at primary tumour diagnosis was 60.8 (IQR 25–75% = 51.8–69.0) years, and median age at PM was 64.5 (IQR 25–75% = 55.1–72.5) years. Median disease-free interval (DFI) was 26.0 (IQR 25–75% = 14.0–45.0) months. Male/female ratio was 1.32:1. Median length of follow-up was 6.1 (IQR 25–75% = 4.2–8.1) years. ARs were performed in 376 patients (22.8%), while nARs were done in 1271 patients (77.2%) (Table 1).

The characteristics of the unmatched cohort are shown in Appendix D (Table A3, Table A4, Table A5, Table A6 and Table A7). Notably, ARs were more frequently associated with open procedures (48.1% versus 41.8%, *p* = 0.029), robotic-assisted thoracoscopic surgery (5.1% versus 2.3%, *p* = 0.005), and hilar-mediastinal lymph node dissection (88.3% versus 27.5%, *p* < 0.001), while nARs were more frequently associated with bilateral metastases (13.1% versus 0.8%, *p* < 0.001) and video-assisted thoracoscopic surgery (VATS) (57.3% versus 46.8%, *p* < 0.001). The prevalence of synchronous/metachronous lung metastases did not significantly differ between groups (*p* = 0·668). Single lung metastases were reported in 69.6% of cases (83.0% in the AR group versus 65.7% in the nAR group, *p* < 0.0001). The OS of the entire cohort at 1, 5, and 10 years was 94.2%, 62.0%, and 41.7%, respectively (Table 2).

Tumour progression was the reported cause of death for 84.0% patients. OS between synchronous and metachronous disease did not differ (*p* = 0.135). The univariable Cox regression did not detect any differences in OS between ARs and nARs (HR = 1.145, 95% CI = 0.954–1.374, *p* = 0.1471). Since the propensity hazard assumption was rejected (*p* < 0.001), the effect of the type of resection on OS was allowed to be time-varying. The multivariable Cox regression model showed that patients in the AR group had significantly worse OS in the first 2.5 years following PM (HR = 1.656, 95% CI = 1.266–2.167, *p* < 0.001), as opposed to after 2.5 years following PM (HR = 0.780, 95% CI = 0.595–1.024, *p* = 0.073). Lobectomies/bilobectomies showed a significantly worse OS than segmentectomies (*p* = 0.002) and wedge resections (*p* = 0.009), while OS was comparable between anatomical segmentectomies and wedge resections (*p* = 0.291). There were no significant differences in OS between ARs and nARs depending on the number of confirmed metastases (*p* = 0.709), and the size of the largest confirmed metastasis (*p* = 0.458). Any-site RFS at 1, 5, and 10 years was 64.1%, 29.6%, and 21.7%, respectively (Table 2). The univariable Cox regression showed no significant association with the broadness of resection margins (HR = 0.991, CI = 0.979–1.003, *p* = 0.128). ARs were associated with a significantly longer any-site RFS (HR = 0.779, 95% CI = 0.667–0.911, *p* = 0.002), which was confirmed at multivariable analyses (HR = 0.789, 95% CI = 0.668–0.932, *p* = 0.005). Locoregional RFS at 1, 5, and 10 years was 74.4%, 42.1%, and 31.3%, respectively (Table 2). A significant correlation with the broadness of resection margins was observed (HR = 0.989, CI = 0.979–1.000, *p* = 0.049). The univariable analysis showed longer locoregional RFS after ARs (HR = 0.643, 95% CI = 0.530–0.779, *p* < 0.0001), which was confirmed at multivariable analyses (HR = 0.655, 95% CI = 0.533–0.804, *p* < 0.0001). In a subset of patients who underwent lymph node dissection (323 in the AR group, 314 in the nAR group), the proportional hazard assumption was rejected (*p* = 0.014), and ARs were associated with a worse OS in the first 2.5 years after PM (HR = 1.540, 95% CI = 1.072–2.212, *p* = 0.020). In the multivariable Cox regression model, the effect of ARs on OS in the first 2.5 years following PM was not significant (HR = 1.393, 95% CI = 0.942–2.060, *p* = 0.097). In the nAR group, OS did not differ between patients who underwent lymph node dissection and those who did not (*p* = 0.154). Site-specific and histology-specific OS and RFS are reported in Appendix E (Table A8 and Table A9).

### 3.2. Matched Cohort Analyses

**Figure 1:**
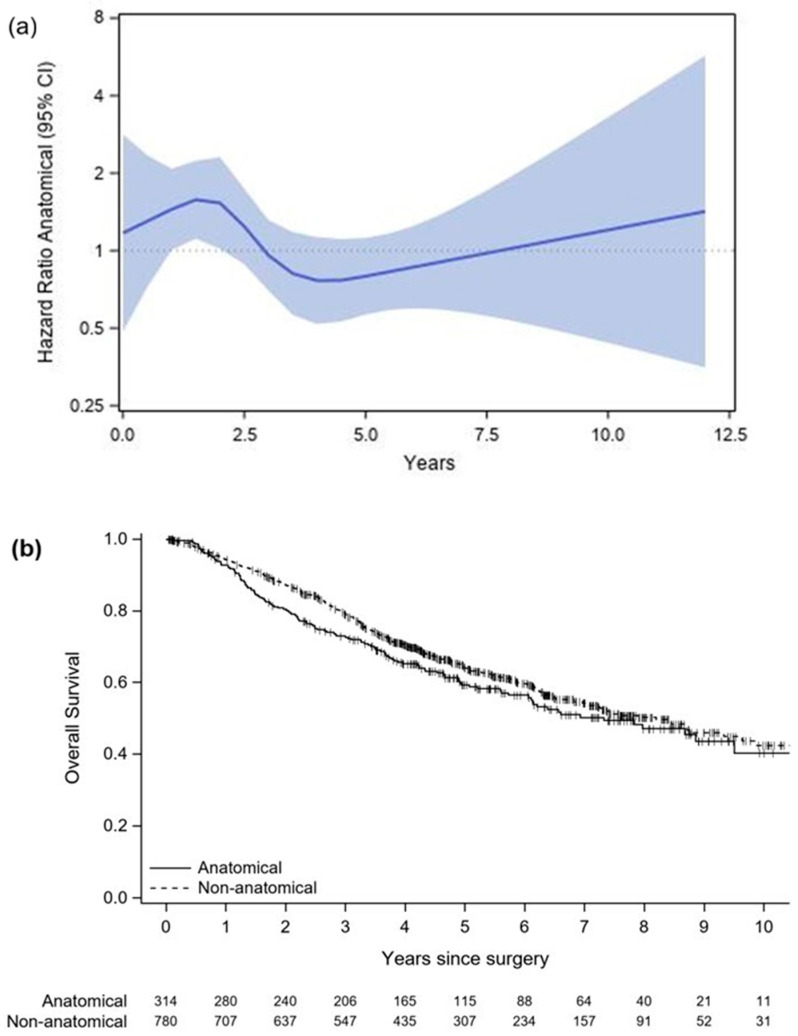
Overall survival in the matched cohort. (**a**) Hazard ratio (95% confidence interval) from a Cox regression with restricted cubic splines allowing the effect of anatomical resection to vary over time. Of note, confidence intervals for the spline-based hazard ratio widen after 10 years, reflecting the limited number of participants remaining at risk. (**b**) Kaplan–Meier curve showing overall survival in the matched cohort.

The two groups were matched 3:1 on age at PM, sex, size of the (largest) metastasis, number of confirmed metastases, and induction therapy before PM. An exact match was required for sex, size of the (largest) metastasis, number of confirmed metastases, and induction therapy before PM (standardised difference was 0). The evaluation of the balance of the variables involved in matching is shown in Appendix F (Table A10). Concerning age at PM, the standardised difference after matching was 0.154 (difference of 1.7 years). After matching, the AR group included 324 patients, while the nAR group consisted of 830 patients (62, 18, and 244 AR patients were matched with 1, 2, and 3 nAR patients, respectively). Information on OS was available for 314 AR and 780 nAR patients. There was no significant difference in OS between ARs and nARs (HR = 1.122, 95% CI = 0.909–1.385, *p* = 0.283). The proportional hazard assumption was rejected (*p* = 0.003). ARs had a worse OS in the first 2.5 years following PM (HR = 1.549, 95% CI = 1.135–2.114, *p* = 0.006) (Figure 1).

**Figure 2:**
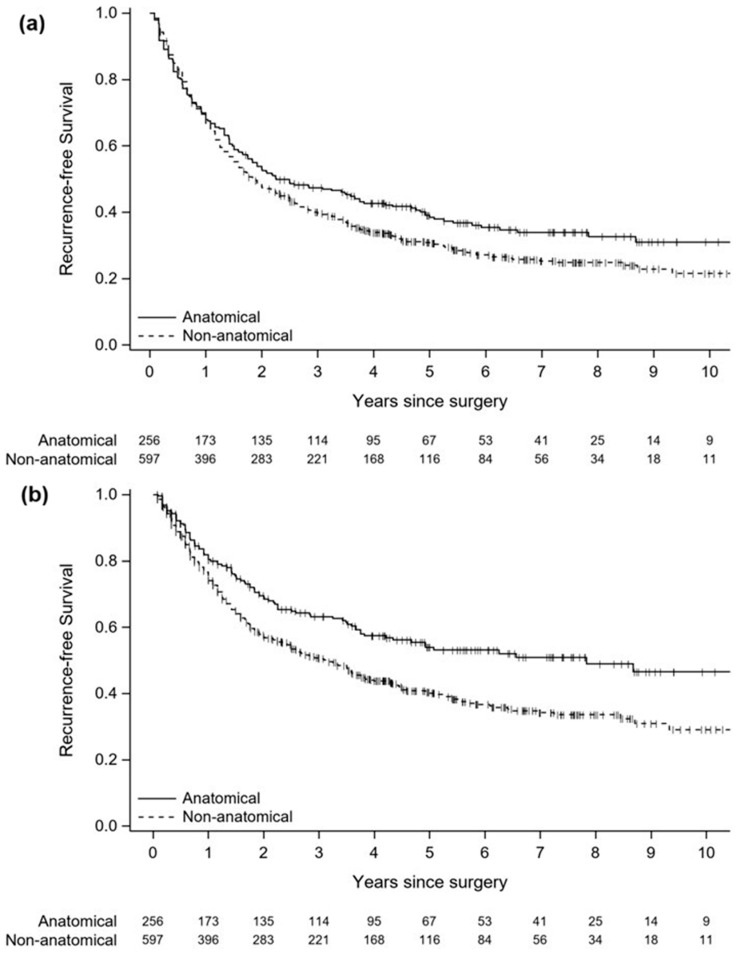
Recurrence-free survival in the matched cohort. (**a**) Any-site recurrence-free survival. (**b**) Locoregional recurrence-free survival.

Information on recurrence was available for 256 patients in the AR group and 597 patients in the nAR group. There was a tendency towards longer any-site RFS after AR, but the difference was not significant (HR = 0.832, 95% CI = 0.690–1.002, *p* = 0.053). Locoregional RFS was significantly longer after AR (HR = 0.651, 95% CI = 0.520–0.817, *p* < 0.001) (Figure 2).

Data on 30-day postoperative complications were available for 324 patients who underwent AR and 830 who underwent nAR (Table 3).

## 4. Discussion

Pulmonary metastases are relatively common findings in patients with extrathoracic malignancies [4,5,6]. PM aims at achieving complete resection of lung metastases and has earned a place among the potentially curative approaches for stage IV tumours. Survival analyses comparing surgical resection to other treatment options for lung metastases are scarce, and the actual benefits of PM remain unclear. In a randomised controlled trial on patients with pulmonary metastases from colorectal cancer, survival rates after PM were similar to those of the control group [24]. However, despite not being supported by high-quality evidence [4], PM is increasingly performed in selected subsets of patients with lung metastases from various primary tumours [7]. This multicentre study assessed the survival impact of the extent of surgical resection in patients undergoing curative-intent PM. The unmatched cohort consisted of more than 1600 patients from 15 European centres, with a follow-up of 6 years and a variety of primary tumours and clinical presentations, being representative of the real-world PM population. After propensity-matching on five clinically relevant variables, no significant OS difference was observed between nARs and ARs. Our findings add to the growing evidence that sublobar lung resections provide equivalent long-term survival benefits to major anatomical resections [13,14]. In addition, our analyses revealed that nARs resulted in comparable OS to ARs regardless of the number of metastases, size of the largest metastasis, and lymph node dissection. Furthermore, we demonstrated that patients undergoing ARs had a worse OS in the early postoperative period (2.5 years). This could be ascribed to several, non-mutually exclusive factors. First, ARs were associated with a greater operative extent, with approximately 70% of procedures consisting of lobectomies or more extensive resections. Second, the AR group showed higher postoperative morbidity, particularly cardiac and respiratory complications and a higher incidence of Clavien–Dindo grade III–V events, which may contribute to early mortality. Third, confounding by indication cannot be excluded, as ARs are typically performed when lesions are central, larger, or technically unsuitable for limited resections. Unfortunately, information on the intraparenchymal location of metastases was not available in our dataset, preventing further exploration of this aspect. Previous studies have shown more severe and long-term invalidating sequelae after ARs, especially after major anatomical resections [25]. However, in this study, when restricting the comparison to anatomical segmentectomy versus wedge resection, OS was comparable, and the survival difference observed in the early period after PM was not detected anymore. Survival rates were remarkably higher than in previously reported cohorts: 5- and 10-year OS were 62.0% (versus 20.0–48.0%) and 41.7% (versus 15.8–37.7%), respectively [5]. This could be attributed to the improvements of systemic treatments for stage IV disease and our strict inclusion criteria (macroscopic complete resection of all lung metastases, no pneumonectomies). Regarding locoregional recurrence, ARs resulted in a significantly longer RFS. This could be related to the broader resection margins in the AR group. In fact, in the unmatched cohort, we observed a significant correlation between the broadness of resection margins and locoregional RFS, in line with previous research showing that narrow resection margins are a risk factor for local recurrences [3,11].

The improvement in loco-regional RFS, however, did not translate into a measurable survival benefit. This likely reflects the fact that overall survival is determined by multiple factors beyond local disease control, including tumour biology, the extent of systemic disease, and the effectiveness of systemic therapies. In the literature, repeated PMs are considered a safe and reliable approach to recurrent lung metastases [3,26]. In our cohort, almost one third of the patients underwent repeated PMs, the majority (86.0%) of them following a nAR as first PM. This may indicate greater preservation of lung parenchyma after the first PM in the nAR group, although differences in multidisciplinary decision-making after prior ARs may also have contributed. The role of lymph node assessment and its impact on OS remains controversial: despite being recommended [4,7,10], nodal dissection is not routinely performed in the setting of PM [8,9,10]. In our cohort, it was performed in 41.4% of cases and was strongly associated with ARs, but, in accordance with the existing research, it did not significantly affect survival after PM [27]. In order to increase the robustness of our findings, our study cohort was highly selected (patients aged ≥18, with lung metastases being the first and only site of disease relapse from an extrathoracic solid tumour, treated with curative-intent PM, exclusion of pneumonectomies, etc.). Nevertheless, it remains representative of real-world data on PM in Europe: more than half of patients suffered from digestive system tumours (mainly colorectal adenocarcinoma) [28], PMs were most often performed for solitary lung metastases (69.9%), mostly by VATS (54.9%), and mostly through nAR (77.2%) [4,7,9,29,30]. In accordance with previous publications [5,6,8], PM confirms itself as a safe procedure with acceptable intraoperative and postoperative morbidity. It has to be noted, however, that PM outcomes may also depend on tumour histology and the local microenvironment [31,32]. The main limitations of our study lie in its retrospective nature, which may introduce selection bias, and in the variety of practice patterns among the participating centres (patient selection protocols, primary tumours, non-surgical treatment strategies, postoperative surveillance protocols). We addressed the heterogeneity of our population by establishing stringent eligibility criteria and matching on clinically significant variables. Of note, median DFI was longer in the AR group (*p* = 0.005); however, since the difference with the nAR group (5.0 months) was deemed clinically irrelevant, DFI was not included among the confounders. Similarly, primary tumour histology was not included among the confounders; therefore, patients with different primary tumours may have been matched. Furthermore, different primary tumour histologies were regrouped based on their anatomical site. A minor residual discrepancy was observed for uncommon cancer types, including bone, muscle, vessel, and soft tissue tumours, which were slightly more frequent in the nAR group. While these represent a small fraction of the cohort, their unique prognostic and treatment profiles compared with the predominant colorectal histology could have influenced the outcomes. OS as well as RFS could theoretically be affected by lymph node dissection, which was more frequent in the AR group. However, nodal positivity was extremely rare (<1% in both groups) and is therefore unlikely to have influenced survival outcomes. Information on the location of pulmonary lesions within the parenchyma (central versus peripheral) was not available, although it represents a major factor influencing the decision between AR versus nAR and may have contributed to the worse early overall survival observed in the AR group. Patients with missing information (e.g., on recurrence) were excluded, potentially leading to an underestimation of the considered outcome. The strength of this study lies in its multicentre design, real-world approach, and large cohort. It assessed several clinically relevant short- and long-term endpoints. The reported evidence is supported by the consistency of the findings of unmatched and matched analyses.

## 5. Conclusions

Our findings support the long-standing surgical strategy of favouring limited resections whenever technically and oncologically feasible. ARs may be associated with longer RFS and remain a valuable option when nARs are not feasible (central, large and/or multiple ipsilobar lesions) while still ensuring acceptable morbidity and similar oncological outcomes. These results contribute to the standardisation of PM and provide meaningful guidance for multidisciplinary decision-making in the management of lung metastases.

## Figures and Tables

**Table 1:** Description of the type of resection.

Type of Resection		n/N (%)
Anatomical	Lobectomy	242/376 (64.4)
	Bilobectomy	15/376 (4.0)
	Segmentectomy	115/376 (30.6)
	Combined lobectomy/segmentectomy	4/376 (1.1)
Non-anatomical	Wedge resection	1103/1271 (86.8)
	Combined wedge resection/precision tumourectomy	116/1271 (9.1)
	Precision tumourectomy	52/1271 (4.1)

**Table 2:** Kaplan–Meier estimates for overall survival and recurrence-free survival after pulmonary metastasectomy.

Years Since PM	OS (95% CI)	Any-Site RFS (95% CI)	Locoregional RFS (95% CI)
1	94.2 (93.0; 95.2)	64.1 (62.1; 66.1)	74.4 (72.4; 76.3)
2	85.6 (83.9; 87.2)	46.4 (44.6; 48.2)	59.1 (57.1; 61.1)
3	76.7 (74.7; 78.5)	38.4 (36.8; 40.0)	51.4 (49.4; 53.3)
5	62.0 (59.9; 64.0)	29.6 (28.3; 31.0)	42.1 (40.2; 43.9)
10	41.7 (39.1; 44.3)	21.7 (20.4; 23.0)	31.3 (29.3; 33.3)
**Location of recurrences**		**n/N (%)**	
Locoregional recurrence		502/1540 (32.6)	
Distant recurrence		206/1540 (13.4)	
Locoregional and distant recurrence		144/1540 (9.4)	
Unknown		80/1540 (5.2)	

CI: confidence interval; OS: overall survival; PM: pulmonary metastasectomy; RFS: recurrence-free survival.

**Table 3:** Postoperative complications in the matched and weighted cohort.

Complications Occurring Within 30 Days from PM	ARs, n/N (%)	nARs, n/N (%)	*p*-Value
Fever > 38 °C	12.0/324 (3.7)	18.8/830 (2.3)	0.202
Atrial fibrillation and/or dysrhythmias	12.0/324 (3.7)	14.1/830 (1.7)	0.064
Myocardial ischemia/infarction	1.0/324 (0.3)	0.0/830 (0.0)	0.109
Thromboembolism	2.0/324 (0.6)	0.9/830 (0.1)	0.095
Prolonged air leaks (>5 days)	19.0/324 (5.9)	17.9/830 (2.2)	0.004
Atelectasis and/or pneumonia	13.0/324 (4.0)	23.1/830 (2.8)	0.313
Respiratory failure	3.0/324 (0.9)	2.6/830 (0.3)	0.156
Broncho-pleural fistula with/without empyema	2.0/324 (0.6)	1.7/830 (0.2)	0.248
Chylothorax	3.0/324 (0.9)	1.7/830 (0.2)	0.070
Vocal fold dysfunction	5.0/324 (1.5)	0.9/830 (0.1)	0.001
Anaemia requiring blood transfusions and/or haemothorax	6.0/324 (1.9)	19.2/830 (2.3)	0.642
Gastro-intestinal complications	3.0/324 (0.9)	8.5/830 (1.0)	0.872
Neurological complications	2.0/324 (0.6)	0.9/830 (0.1)	0.095
Urogenital complications	2.0/324 (0.6)	10.7/830 (1.3)	0.353

AR: anatomical resection; nAR: non-anatomical resection; PM: pulmonary metastasectomy. *p*-values are based on a Chi^2^ test. ARs were associated with a higher rate of complications (22.2% versus 13.7%, *p* = 0.001). In a subgroup of 176 matched patients who experienced postoperative complications, ARs showed a higher prevalence of Clavien-Dindo grade III-IV-V morbidity (27.8% versus 12.0%, *p* = 0.010).

## Data Availability

The data presented in this study are available on request from the corresponding author due to ethical reasons.

## Abbreviations

The following abbreviations are used in this manuscript:

AR	anatomical resection
ASA	American Society of Anesthesiologists
CI	confidence interval
DFI	disease-free interval
HR	hazard ratio
IQR	interquartile range
nAR	non-anatomical resection
OS	overall survival
PM	pulmonary metastasectomy
REDCap	Research Electronic Data Capture
RFS	recurrence-free survival
SD	standard deviation
STROBE	Strengthening the Reporting of Observational Studies in Epidemiology
VATS	video-assisted thoracoscopic surgery

## Appendix A

**Table A1:** STROBE Statement—Checklist of items that should be included in reports of cohort studies.

	Item No	Recommendation	Page No
**Title and abstract**	1	(a) Indicate the study’s design with a commonly used term in the title or the abstract	1, 2
		(b) Provide in the abstract an informative and balanced summary of what was done and what was found	2
**Introduction**			
**Background/rationale**	2	Explain the scientific background and rationale for the investigation being reported	2–3
**Objectives**	3	State specific objectives, including any prespecified hypotheses	2–3
**Methods**			
**Study design**	4	Present key elements of study design early in the paper	3–5
**Setting**	5	Describe the setting, locations, and relevant dates, including periods of recruitment, exposure, follow-up, and data collection	3–5
**Participants**	6	(a) Give the eligibility criteria, and the sources and methods of selection of participants. Describe methods of follow-up	3–5
		(b) For matched studies, give matching criteria and number of exposed and unexposed	3–5
**Variables**	7	Clearly define all outcomes, exposures, predictors, potential confounders, and effect modifiers. Give diagnostic criteria, if applicable	3–5
**Data sources/measurement**	8	For each variable of interest, give sources of data and details of methods of assessment (measurement). Describe comparability of assessment methods if there is more than one group	3–5
**Bias**	9	Describe any efforts to address potential sources of bias	3–5
**Study size**	10	Explain how the study size was arrived at	3–5
**Quantitative variables**	11	Explain how quantitative variables were handled in the analyses. If applicable, describe which groupings were chosen and why	3–5
**Statistical methods**	12	(a) Describe all statistical methods, including those used to control for confounding	3–5
		(b) Describe any methods used to examine subgroups and interactions	
		(c) Explain how missing data were addressed	
		(d) If applicable, explain how loss to follow-up was addressed	
		(e) Describe any sensitivity analyses	
**Results**			
**Participants**	13	(a) Report numbers of individuals at each stage of study—e.g., numbers potentially eligible, examined for eligibility, confirmed eligible, included in the study, completing follow-up, and analysed	5, Appendix C
		(b) Give reasons for non-participation at each stage	
		(c) Consider use of a flow diagram	
**Descriptive data**	14	(a) Give characteristics of study participants (e.g., demographic, clinical, social) and information on exposures and potential confounders	5–9, Appendix D
		(b) Indicate number of participants with missing data for each variable of interest	
		(c) Summarise follow-up time (e.g., average and total amount)	
**Outcome data**	15	Report numbers of outcome events or summary measures over time	Figure 1 and Figure 2, Appendix D and Appendix F
**Main results**	16	(a) Give unadjusted estimates and, if applicable, confounder-adjusted estimates and their precision (e.g., 95% confidence interval). Make clear which confounders were adjusted for and why they were included	5–9, Appendix F
		(b) Report category boundaries when continuous variables were categorised	
		(c) If relevant, consider translating estimates of relative risk into absolute risk for a meaningful time period	
**Other analyses**	17	Report other analyses done—e.g., analyses of subgroups and interactions, and sensitivity analyses	5–9, Appendix D
**Discussion**			
**Key results**	18	Summarise key results with reference to study objectives	9–11
**Limitations**	19	Discuss limitations of the study, taking into account sources of potential bias or imprecision. Discuss both direction and magnitude of any potential bias	9–11
**Interpretation**	20	Give a cautious overall interpretation of results considering objectives, limitations, multiplicity of analyses, results from similar studies, and other relevant evidence	9–11
**Generalisability**	21	Discuss the generalisability (external validity) of the study results	9–11
**Other information**			
**Funding**	22	Give the source of funding and the role of the funders for the present study and, if applicable, for the original study on which the present article is based	11

## Appendix B

List of European centres participating to the study (IEO 1438—13 January 2021)

1. Divisione di Chirurgia Toracica, Istituto Europeo di Oncologia, Milano, Italy;
2. Divisione Universitaria di Chirurgia Toracica, Azienda Ospedaliero-Universitaria Città della Salute e della Scienza, Torino, Italy;
3. Unità Operativa di Chirurgia Toracica, Azienda Ospedaliero-Universitaria Pisana, Pisa, Italy;
4. Unità Operativa Complessa di Chirurgia Toracica, Azienda Ospedaliero-Universitaria Consorziale Policlinico, Bari, Italy;
5. Unità Operativa Complessa di Chirurgia Toracica, Ospedale Policlinico Universitario Agostino Gemelli, Roma, Italy;
6. Service de Chirurgie thoracique et des maladies de l’œsophage, Assistance Publique Hôpitaux de Marseille, Marseille, France;
7. Service de Chirurgie Thoracique et Cardio-Vasculaire, Centre Hospitalier Universitaire, Rouen, France;
8. Service de Chirurgie Cardio-Thoracique, Centre Hospitalier Universitaire de Limoges, Limoges, France;
9. Service de Chirurgie Thoracique et Cervicale et Transplantation Pulmonaire, Centre Hospitalier Universitaire de Bordeaux, Pessac, France;
10. Servicio de Cirugía Torácica, Complejo Asistencial Universitario de Salamanca, Salamanca, Spain;
11. Klinik für Thoraxchirurgie, Universitätsspital Zürich, Zürich, Switzerland;
12. Service de Chirurgie Thoracique, Centre Hospitalier Universitaire Vaudois, Lausanne, Switzerland;
13. Department of Thoracic Surgery, Marmara University Faculty of Medicine, Istanbul, Turkey;
14. Afdeling for Hjerte- og Lungekirurgi, Rigshospitalet og Institut for Klinisk Medicin, Københavns Universitet, København, Denmark;
15. Thoraxheelkunde, Universitaire Ziekenhuizen Leuven, Leuven, Belgium.

## Appendix C

**Table A2:** Reasons for exclusion from analyses.

Reason for Exclusion	N
Both anatomical and non-anatomical resections during the same procedure	132
Subjects not fulfilling eligibility criteria	53
Inconsistencies	36
Missing information on type of resection	7

## Appendix D

**Table A3:** Demographic and baseline characteristics of the unmatched cohort.

Variable	Statistic	ARs	nARs	*p*-Value
**Sex**				0.223
Female	n/N (%)	152/376 (40.4)	558/1269 (44.0)	
Male	n/N (%)	224/376 (59.6)	711/1269 (56.0)	
**Age at primary diagnosis** (**years**)	N	368	1247	0.033
	Mean (SD)	61.0 (11.5)	59.1 (13.3)	
	Median (IQR)	61.7 (53.8; 69.4)	60.5 (51.3; 68.8)	
	Range	(15.9; 87.8)	(0.0; 85.8)	
**DFI** (**months**)	N	292	974	0.005
	Mean (SD)	43.4 (46.7)	35.9 (41.4)	
	Median (IQR)	30.0 (16.0; 52.0)	25.0 (14.0; 43.0)	
	Range	(0.0; 406.0)	(0.0; 601.0)	
**Age at PM** (**years**)	N	376	1271	0.003
	Mean (SD)	64.9 (11.2)	62.3 (12.9)	
	Median (IQR)	66.2 (57.3; 73.3)	64.0 (54.4; 72.3)	
	Range	(26.2; 91.0)	(18.6; 88.3)	
**Timing of PM**				0.668
Synchronous disease	n/N (%)	29/353 (8.2)	89/1183 (7.5)	
Metachronous disease	n/N (%)	324/353 (91.8)	1094/1183 (92.5)	
**Year of surgery** (**first PM if bilateral**)				0.566
2010	n/N (%)	27/376 (7.2)	78/1271 (6.1)	
2011	n/N (%)	21/376 (5.6)	94/1271 (7.4)	
2012	n/N (%)	33/376 (8.8)	106/1271 (8.3)	
2013	n/N (%)	41/376 (10.9)	125/1271 (9.8)	
2014	n/N (%)	37/376 (9.8)	145/1271 (11.4)	
2015	n/N (%)	49/376 (13.0)	183/1271 (14.4)	
2016	n/N (%)	49/376 (13.0)	178/1271 (14.0)	
2017	n/N (%)	50/376 (13.3)	180/1271 (14.2)	
2018	n/N (%)	69/376 (18.4)	182/1271 (14.3)	
**Treatment of the primary tumour**	n/N (%)	369/375 (98.4)	1261/1270 (99.3)	0.111
**Completeness of resection**				0.168
R0	n/N (%)	317/328 (96.6)	1000/1055 (94.8)	
R1	n/N (%)	11/328 (3.4)	55/1055 (5.2)	
**Primary site**				0.015
Head and Neck	n/N (%)	15/376 (4.0)	84/1271 (6.6)	
Digestive system	n/N (%)	218/376 (58.0)	653/1271 (51.4)	
Urogenital/male reproductive system	n/N (%)	50/376 (13.3)	174/1271 (13.7)	
Breasts/female reproductive system	n/N (%)	46/376 (12.2)	139/1271 (10.9)	
Skin	n/N (%)	28/376 (7.5)	96/1271 (7.6)	
Nervous system	n/N (%)	1/376 (0.3)	4/1271 (0.3)	
Bone, muscle, vessel, and soft tissue	n/N (%)	12/376 (3.2)	106/1271 (8.3)	
Other	n/N (%)	6/376 (1.6)	15/1271 (1.2)	
**Histology**				0.027
Adenocarcinoma	n/N (%)	253/375 (67.5)	748/1267 (59.0)	
Squamous cell carcinoma	n/N (%)	16/375 (4.3)	61/1267 (4.8)	
Sarcoma	n/N (%)	22/375 (5.9)	137/1267 (10.8)	
Germ cell tumour	n/N (%)	2/375 (0.5)	10/1267 (0.8)	
Melanoma	n/N (%)	29/375 (7.7)	95/1267 (7.5)	
Other	n/N (%)	53/375 (14.1)	216/1267 (17.1)	

AR: anatomical resection; DFI: disease-free interval; IQR: interquartile range; nAR: non-anatomical resection; PM: lung metastasectomy; SD: standard deviation.

**Table A4:** Preoperative characteristics of the unmatched cohort.

Variable	Statistic	ARs	nARs	*p*-Value
**Major comorbidities at the time of PM**	n/N (%)	198/374 (52.9)	685/1267 (54.0)	0.702
Atrial fibrillation and/or dysrhythmias	n/N (%)	28/375 (7.5)	70/1271 (5.5)	0.159
Myocardial ischemia and/or infarction	n/N (%)	23/375 (6.2)	68/1271 (5.4)	0.560
Carotid artery stenosis and/or cerebrovascular events	n/N (%)	15/375 (4.0)	55/1271 (4.3)	0.783
Heart valve disease	n/N (%)	14/375 (3.7)	34/1271 (2.7)	0.284
Thromboembolism and/or chronic obliterative vascular disease	n/N (%)	23/375 (6.1)	68/1271 (5.4)	0.560
Heart failure	n/N (%)	9/375 (2.4)	17/1271 (1.3)	0.147
Chronic obstructive pulmonary disease	n/N (%)	16/375 (4.3)	88/1271 (6.9)	0.063
Asthma and/or obstructive sleep apnoea syndrome	n/N (%)	12/375 (3.2)	32/1271 (2.5)	0.472
Pulmonary hypertension	n/N (%)	1/375 (0.3)	3/1271 (0.2)	0.916
Pulmonary fibrosis	n/N (%)	1/375 (0.3)	2/1271 (0.2)	0.663
Diabetes	n/N (%)	42/375 (11.2)	126/1271 (9.9)	0.470
Obesity	n/N (%)	36/375 (9.6)	64/1271 (5.0)	0.001
Dyslipidaemia	n/N (%)	47/375 (12.5)	179/1271 (14.1)	0.443
Hypoalbuminemia and/or liver disease	n/N (%)	7/375 (1.9)	31/1271 (2.4)	0.517
Other cancer (s)	n/N (%)	32/375 (8.5)	112/1271 (8.8)	0.867
**ASA score**				0.653
1	n/N (%)	15/370 (4.1)	60/1249 (4.8)	
2	n/N (%)	205/370 (55.4)	647/1249 (51.8)	
3	n/N (%)	146/370 (39.5)	529/1249 (42.4)	
4	n/N (%)	4/370 (1.1)	13/1249 (1.0)	
**FEV1** (**litres**)	N	263	827	0.204
	Mean (SD)	2.7 (0.8)	2.7 (0.8)	
	Median (IQR)	2.5 (2.0; 3.1)	2.7 (2.1; 3.2)	
	Range	(0.5; 4.7)	(0.5; 5.8)	
**DLCO** (**%**)	N	265	725	0.939
	Mean (SD)	86.9 (18.9)	87.0 (19.8)	
	Median (IQR)	86.0 (74.3; 99.0)	86.0 (74.6; 99.0)	
	Range	(36.0; 139.2)	(32.7; 154.0)	
**Induction therapy before PM**	n/N (%)	49/375 (13.1)	105/1271 (8.3)	0.00
Chemotherapy	n/N (%)	42/375 (11.2)	98/1271 (7.7)	0.033
Radiation therapy	n/N (%)	6/375 (1.6)	5/1271 (0.4)	0.012
Hormone therapy/immunotherapy/targeted therapy	n/N (%)	4/375 (1.1)	9/1271 (0.7)	0.491
Treatment interruption due to toxicity	n/N (%)	1/375 (0.3)	1/1271 (0.1)	0.358

AR: anatomical resection; ASA: American Society of Anaesthesiologists; DLCO: diffusing lung capacity of carbon monoxide; FEV1: forced expiratory volume in one second; IQR: interquartile range; nAR: non-anatomical resection; PM: lung metastasectomy; SD: standard deviation.

**Table A5:** Surgical characteristics of the unmatched cohort.

Variable	Statistic	ARs	nARs	*p*-Value
**Side of surgery**				<0.001
Right	n/N (%)	214/376 (56.9)	602/1271 (47.4)	
Left	n/N (%)	159/376 (42.3)	503/1271 (39.6)	
Bilateral	n/N (%)	3/376 (0.8)	166/1271 (13.1)	
**Type of bilateral PM**				0.848
Concurrent	n/N (%)	1/3 (33.3)	47/169 (28.3)	
Sequential	n/N (%)	2/3 (66.7)	119/169 (71.7)	
**Type of surgical approach**				
VATS	n/N (%)	176/376 (46.8)	728/1271 (57.3)	<0.001
RATS	n/N (%)	19/376 (5.1)	29/1271 (2.3)	0.005
Open	n/N (%)	181/376 (48.1)	531/1271 (41.8)	0.029
**Location of the resected lung lesions**				<0.001
Lower lobe (s)	n/N (%)	163/369 (44.2)	441/1264 (34.9)	
Middle-Upper lobe (s)	n/N (%)	192/369 (52.0)	461/1264 (36.5)	
Both	n/N (%)	14/369 (3.8)	362/1264 (28.6)	
**Total number of resected lung lesions**	N	340	1115	<0.001
	Mean (SD)	1.3 (0.8)	2.7 (4.2)	
	Median (IQR)	1.0 (1.0; 1.0)	1.0 (1.0; 3.0)	
	Range	(1.0; 6.0)	(1.0; 57.0)	
**Hilar-mediastinal lymph node dissection**	n/N (%)	332/376 (88.3)	349/1271 (27.5)	<0.001
**Intraoperative adverse events**	n/N (%)	4/376 (1.1)	5/1271 (0.4)	0.121

AR: anatomical resection; IQR: interquartile range; nAR: non-anatomical resection; PM: lung metastasectomy; RATS: robot-assisted thoracoscopic surgery; SD: standard deviation; VATS: video-assisted thoracoscopic surgery.

**Table A6:** Histopathologic characteristics of the unmatched cohort.

Variable	Statistic	ARs	nARs	*p*-Value
**Number of metastases**				<0.001
1	n/N (%)	312/376 (83.0)	835/1271 (65.7)	
2–4	n/N (%)	57/376 (15.2)	335/1271 (26.4)	
>4	n/N (%)	5/376 (1.3)	97/1271 (7.6)	
Unknown	n/N (%)	2/376 (0.5)	4/1271 (0.3)	
**Size of largest metastasis** (**mm**)				<0.001
<10	n/N (%)	34/369 (9.2)	301/1241 (24.2)	
10–30	n/N (%)	237/369 (64.2)	858/1241 (69.1)	
>30	n/N (%)	98/369 (26.6)	82/1241 (6.6)	
**Negative resection margin** (**mm**)	N	200	715	<0.001
	Mean (SD)	16.6 (14.0)	6.7 (6.1)	
	Median (IQR)	11.0 (6.0; 25.0)	5.0 (2.0; 10.0)	
	Range	(0.0; 60.0)	(0.0; 40.0)	
**Completeness of resection**				0.418
R0	n/N (%)	331/376 (88.0)	1080/1271 (85.5)	
R1	n/N (%)	12/376 (3.2)	51/1271 (4.0)	
Unknown	n/N (%)	33/343 (8.8)	140/1271 (11.0)	

AR: anatomical resection; IQR: interquartile range; nAR: non-anatomical resection; SD: standard deviation.

**Table A7:** Postoperative course of the unmatched cohort.

Variable	Statistic	ARs	nARs	*p*-Value
**ICU stay longer than 24 h**	n/N (%)	16/370 (4.3)	22/1242 (1.8)	0.004
**Complications within 30 days from PM**	n/N (%)	83/376 (22.1)	156/1271 (12.3)	<0.001
Fever > 38 °C	n/N (%)	14/376 (3.7)	23/1271 (1.8)	0.027
Atrial fibrillation and/or dysrhythmias	n/N (%)	13/376 (3.5)	16/1271 (1.3)	0.004
Myocardial ischemia/infarction	n/N (%)	2/376 (0.5)	0/1271 (0.0)	0.009
Thromboembolism	n/N (%)	2/376 (0.5)	4/1271 (0.3)	0.537
Prolonged air leaks (>5 days)	n/N (%)	21/376 (5.6)	26/1271 (2.1)	<0.001
Atelectasis and/or pneumonia	n/N (%)	17/376 (4.5)	29/1271 (2.3)	0.020
Respiratory failure	n/N (%)	3/376 (0.8)	5/1271 (0.4)	0.319
Broncho-pleural fistula with or without empyema	n/N (%)	2/376 (0.5)	3/1271 (0.2)	0.358
Chylothorax	n/N (%)	3/376 (0.8)	2/1271 (0.2)	0.047
Vocal fold dysfunction	n/N (%)	6/376 (1.6)	1/1271 (0.1)	<0.001
Anaemia requiring blood transfusions and/or haemothorax	n/N (%)	8/376 (2.1)	26/1271 (2.1)	0.915
Gastro-intestinal complications	n/N (%)	3/376 (0.8)	11/1271 (0.9)	0.904
Neurological complications	n/N (%)	2/376 (0.5)	3/1271 (0.2)	0.358
Urogenital complications	n/N (%)	2/376 (0.5)	14/1271 (1.1)	0.325
**Complication grading according to Clavien-Dindo classification**				0.448
I	n/N (%)	31/83 (37.4)	57/155 (36.8)	
II	n/N (%)	31/83 (37.4)	70/155 (45.2)	
III	n/N (%)	17/83 (20.5)	23/155 (14.9)	
IV	n/N (%)	3/83 (3.6)	5/155 (3.2)	
V	n/N (%)	1/83 (1.2)	0/155 (0.0)	
**Length of postoperative stay**	N	373	1155	<0.001
	Mean (SD)	6.6 (4.9)	4.5 (3.0)	
	Median (IQR)	5.0 (4.0; 7.0)	4.0 (3.0; 5.0)	
	Range	(1.0; 43.0)	(0.0; 32.0)	

AR: anatomical resection; ICU: intensive care unit; IQR: interquartile range; nAR: non-anatomical resection; PM: lung metastasectomy; SD: standard deviation.

## Appendix E

**Table A8:** Kaplan–Meier estimates and hazard ratios (anatomical versus non-anatomical resections) for overall survival and recurrence-free survival after pulmonary metastasectomy as a function of primary tumour site.

**Primary Tumour Site**	**Overall Survival**					
	**5-Year Estimates (95% CI)**		**Univariable Analysis †**		**Multivariable Analysis ₹**	
	**ARs**	**nARs**	**HR (95% CI)**	***p*-Value**	**HR (95% CI)**	***p*-Value**
Head and neck	70.7 (39.4; 87.9)	52.0 (39.1; 63.5)	0.533 (0.189; 1.505)	0.235	*	*
Digestive system	57.8 (50.3; 64.6)	67.2 (63.0; 71.0)	1.260 (0.991; 1.602)	0.059	1.177 (0.907; 1.528)	0.219
Urogenital/male reproductive system	53.2 (37.6; 66.5)	68.1 (59.7; 75.1)	1.574 (0.992; 2.497)	0.054	1.999 (1.147; 3.482)	0.015
Breast/female reproductive system	63.6 (45.0; 77.3)	56.8 (46.6; 65.8)	1.056 (0.611; 1.824)	0.845	1.215 (0.664; 2.224)	0.528
Skin tumours, nervous system, and others	63.6 (44.9; 77.5)	51.4 (41.3; 60.5)	0.785 (0.445; 1.385)	0.403	0.879 (0.461; 1.677)	0.695
Bone, muscle, vessels, and soft tissues	58.3 (27.0; 80.1)	55.7 (43.6; 66.1)	1.285 (0.568; 2.906)	0.547	*	*
**Primary Tumour Site**	**Locoregional Recurrence-Free Survival**					
	**5-Year Estimates (95% CI)**		**Univariable Analysis †**		**Multivariable Analysis ₹**	
	**ARs**	**nARs**	**HR (95% CI)**	***p*-Value**	**HR (95% CI)**	***p*-Value**
Head and neck	72.7 (37.1; 90.3)	16.5 (7.8; 27.9)	0.193 (0.060; 0.620)	0.006	*	*
Digestive system	54.1 (45.5; 61.9)	46.7 (42.0; 51.3)	0.747 (0.579; 0.964)	0.025	0.680 (0.515; 0.899)	0.007
Urogenital/male reproductive system	50.9 (32.6; 66.6)	35.2 (26.5; 43.9)	0.762 (0.453; 1.282)	0.306	0.857 (0.475; 1.546)	0.608
Breast/female reproductive system	47.2 (28.2; 64.0)	27.6 (18.6; 37.3)	0.527 (0.305; 0.909)	0.021	0.481 (0.255; 0.909)	0.024
Skin tumours, nervous system, and others	31.5 (11.4; 54.2)	33.9 (22.7; 45.5)	0.944 (0.520; 1.715)	0.851	1.015 (0.477; 2.156)	0.9670
Bone, muscle, vessels, and soft tissues	42.9 (9.8; 73.4)	25.2 (15.5; 36.2)	0.965 (0.349; 2.667)	0.9457	*	*

AR: anatomical resection; CI: confidence interval; HR: hazard ratio; nAR: non-anatomical resection. * No multivariable analysis performed due to small number of observations and/or events. † Results from an univariable Cox regression model assuming proportional hazards. ₹ Results from a multivariable Cox regression model with a random centre effect.

**Table A9:** Kaplan–Meier estimates and hazard ratios (anatomical versus non-anatomical resections) for overall survival and recurrence-free survival after pulmonary metastasectomy as a function of primary tumour histology.

**Primary Tumour Histology**	**Overall Survival**					
	**5-Year Estimates (95% CI)**		**Univariable Analysis †**		**Multivariable Analysis ₹**	
	**ARs**	**nARs**	**HR (95% CI)**	***p*-Value**	**HR (95% CI)**	***p*-Value**
Adenocarcinoma	59.4 (52.5; 65.7)	68.5 (64.7; 72.0)	1.313 (1.049; 1.643)	0.017	1.257 (0.984; 1.607)	0.068
Squamous cell carcinoma	75.0 (46.3; 89.8)	34.7 (21.5; 48.2)	0.346 (0.123; 0.973)	0.044	*	*
Sarcoma	52.4 (29.7; 70.9)	57.7 (47.1; 66.9)	1.493 (0.792; 2.816)	0.216	1.024 (0.474; 2.213)	0.951
Melanoma	68.0 (47.5; 81.9)	46.1 (35.2; 56.4)	0.61 (0.315; 1.164)	0.1324	0.613 (0.284; 1.320)	0.211
**Primary Tumour Histology**	**Locoregional Recurrence-Free Survival**					
	**5-Year Estimates (95% CI)**		**Univariable Analysis †**		**Multivariable Analysis ₹**	
	**ARs**	**nARs**	**HR (95% CI)**	***p*-Value**	**HR (95% CI)**	***p*-Value**
Adenocarcinoma	53.1 (45.2; 60.4)	45.1 (40.7; 49.4)	0.742 (0.587; 0.939)	0.013	0.708 (0.549; 0.914)	0.008
Squamous cell carcinoma	73.8 (24.5; 93.7)	18.7 (7.9; 33.0)	0.103 (0.025; 0.429)	0.002	*	*
Sarcoma	40.4 (15.2; 64.6)	25.1 (16.7; 34.5)	0.749 (0.361; 1.554)	0.437	0.777 (0.348; 1.733)	0.538
Melanoma	37.7 (13.8; 61.8)	35.0 (21.1; 49.3)	0.955 (0.483; 1.888)	0.895	1.135 (0.469; 2.747)	0.779

AR: anatomical resection; CI: confidence interval; HR: hazard ratio; nAR: non-anatomical resection. * No multivariable analysis performed due to small number of observations and/or events. † Results from an univariable Cox regression model assuming proportional hazards. ₹ Results from a multivariable Cox regression model with a random center effect.

## Appendix F

**Table A10:** Evaluation of balance of the variables involved in matching.

Variable	Statistic	ARs	nARs	*p*-Value
**Sex**				0.694
Female	n/N (%)	136/324 (42.0)	359/830 (43.3)	
Male	n/N (%)	188/324 (58.0)	471/830 (56.7)	
**Age at PM** (**years**)	N	324	830	0.030
	Mean (SD)	65.9 (10.7)	64.38 (11.2)	
	Median (IQR)	66.9 (59.4; 73.7)	65.3 (57.6; 72.9)	
**Number of metastases**				
1	n/N (%)	266/324 (82.1)	661/830 (79.6)	#
2–4	n/N (%)	53/324 (16.4)	154/830 (18.6)	0.3822
>4	n/N (%)	5/324 (1.5)	15/830 (1.8)	0.7574
**Size of largest metastasis** (**mm**)				
<10	n/N (%)	32/324 (9.9)	95/830 (11.4)	#
10–30	n/N (%)	234/324 (72.2)	660/830 (79.5)	0.0077
>30	n/N (%)	58/324 (17.9)	75/830 (9.0)	<0.001
**Induction therapy before PM**	n/N (%)	37.0 (11.4)	69.0 (8.3)	0.101

AR: anatomical resection; IQR: interquartile range; nAR: non-anatomical resection; PM: lung metastasectomy; SD: standard deviation; #: reference value.
