# Correction: Prisciandaro et al. Anatomical Versus Non-Anatomical Pulmonary Metastasectomy: European Multicentre Analysis. *Cancers* 2026, *18*, 1037

**DOI:** 10.3390/cancers18101662

**Published:** 2026-05-21

**Authors:** Elena Prisciandaro, Luca Bertolaccini, Steffen Fieuws, Andrea Cara, Lorenzo Spaggiari, Lin Huang, René H. Petersen, Marco Lucchi, Maria G. Mastromarino, Annalisa Barbarossa, Paul De Leyn, Matteo Roffinella, Enrico Ruffini, Abid Donlagic, Michel Gonzalez, Marta G. Fuentes-Gago, Clara Forcada-Barreda, Maria T. Congedo, Stefano Margaritora, Yaniss Belaroussi, Matthieu Thumerel, Jérémy Tricard, Pierre Felix, Nina Lebeda, Isabelle Opitz, Angela De Palma, Giuseppe Marulli, Cesare Braggio, Pascal A. Thomas, Frankie Mbadinga, Jean-Marc Baste, Bihter Sayan, Bedrettin Yildizeli, Jeroen Dekervel, Dirk E. Van Raemdonck, Walter Weder, Laurens J. Ceulemans

**Affiliations:** 1 Department of Thoracic Surgery, University Hospitals of Brussels, 1070 Brussels, Belgium; 2 Department of Medical and Surgical Sciences (DIMEC), University of Bologna, 40126 Bologna, Italy; 3 Department of Thoracic Surgery, IEO, European Institute of Oncology IRCCS, 20141 Milano, Italy; 4 Department of Oncology and Hemato-Oncology, University of Milan, 20122 Milano, Italy; 5 KU Leuven, Interuniversity Institute for Biostatistics and Statistical Bioinformatics, 3000 Leuven, Belgium; 6 Department of Cardiothoracic Surgery, Copenhagen University Hospital, Rigshospitalet, 2100 Copenhagen, Denmark; 7 Division of Thoracic Surgery, Cardiac, Thoracic and Vascular Department, University Hospital of Pisa, 56124 Pisa, Italy; 8 Department of Thoracic Surgery, University Hospitals Leuven, 3000 Leuven, Belgium; 9 KU Leuven, Laboratory of Respiratory Diseases and Thoracic Surgery (BREATHE), CHROMETA, 3000 Leuven, Belgium; 10 Department of Thoracic Surgery, Azienda Ospedaliera Universitaria Città della Salute e della Scienza di Torino, 10126 Torino, Italy; 11 Department of Surgical Science, Section of Thoracic Surgery, University of Torino, 10126 Torino, Italy; 12 Service of Thoracic Surgery, University Hospital of Lausanne, 1011 Lausanne, Switzerland; 13 Department of Thoracic Surgery, Salamanca University Hospital, 37007 Salamanca, Spain; 14 Unit of Thoracic Surgery, Fondazione Policlinico Universitario A. Gemelli IRCCS, 00168 Roma, Italy; 15 Department of Thoracic Surgery, University Hospital Bordeaux, 33604 Pessac, France; 16 Department of Cardiac and Thoracic Surgery, University Hospital Limoges, 87042 Limoges, France; 17 Department of Thoracic Surgery, University Hospital Zürich, 8091 Zürich, Switzerland; 18 Section of Thoracic Surgery, Department of Precision and Regenerative Medicine and Ionian Area, University of Bari “Aldo Moro”, 70124 Bari, Italy; 19 Department of Thoracic Surgery, Lung Transplantation and Oesophageal Diseases, North Hospital, 13015 Marseille, France; 20 Department of General and Cardiothoracic Surgery, University Hospital Rouen, 76031 Rouen, France; 21 Department of Thoracic Surgery, Marmara University School of Medicine, 34854 Istanbul, Turkey; 22 Department of Digestive Oncology, University Hospitals Leuven, 3000 Leuven, Belgium; 23 Department of Thoracic Surgery, Bethanien Klinik, 8044 Zürich, Switzerland

## Addition of Authors

In the original publication [1], the membership of the ESTS Pulmonary Metastasectomy Initiative (Andrea Cara, Lorenzo Spaggiari, Lin Huang, René H. Petersen, Marco Lucchi, Maria G. Mastromarino, Annalisa Barbarossa, Paul De Leyn, Matteo Roffinella, Enrico Ruffini, Abid Donlagic, Michel Gonzalez, Marta G. Fuentes-Gago, Clara Forcada-Barreda, Maria T. Congedo, Stefano Margaritora, Yaniss Belaroussi, Matthieu Thumerel, Jérémy Tricard, Pierre Felix, Nina Lebeda, Isabelle Opitz, Angela De Palma, Giuseppe Marulli, Cesare Braggio, Pascal A. Thomas, Frankie Mbadinga, Jean-Marc Baste, Bihter Sayan, Bedrettin Yildizeli, Jeroen Dekervel, Dirk E. Van Raemdonck, Walter Weder) was not included as co-authors in the original publication, but was instead listed in the Acknowledgments section.

The correct authorship is listed below:

Elena Prisciandaro ^1,2,^*, Luca Bertolaccini ^3,4^, Steffen Fieuws ^5^, Andrea Cara ^3^, Lorenzo Spaggiari ^3,4^, Lin Huang ^6^, René H. Petersen ^6^, Marco Lucchi ^7^, Maria G. Mastromarino ^7^, Annalisa Barbarossa ^8^, Paul De Leyn ^8,9^, Matteo Roffinella ^10^, Enrico Ruffini ^11^, Abid Donlagic ^12^, Michel Gonzalez ^12^, Marta G. Fuentes-Gago ^13^, Clara Forcada-Barreda ^13^, Maria T. Congedo ^14^, Stefano Margaritora ^14^, Yaniss Belaroussi ^15^, Matthieu Thumerel ^15^, Jérémy Tricard ^16^, Pierre Felix ^16^, Nina Lebeda ^17^, Isabelle Opitz ^17^, Angela De Palma ^18^, Giuseppe Marulli ^18^, Cesare Braggio ^19^, Pascal A. Thomas ^19^, Frankie Mbadinga ^20^, Jean-Marc Baste ^20^, Bihter Sayan ^21^, Bedrettin Yildizeli ^21^, Jeroen Dekervel ^22^, Dirk E. Van Raemdonck ^8,9^, Walter Weder ^23^ and Laurens J. Ceulemans ^8,9^

^1^  Department of Thoracic Surgery, University Hospitals of Brussels, 1070 Brussels, Belgium
^2^  Department of Medical and Surgical Sciences (DIMEC), University of Bologna, 40126 Bologna, Italy
^3^  Department of Thoracic Surgery, IEO, European Institute of Oncology IRCCS, 20141 Milano, Italy
^4^  Department of Oncology and Hemato-Oncology, University of Milan, 20122 Milano, Italy
^5^  KU Leuven, Interuniversity Institute for Biostatistics and Statistical Bioinformatics, 3000 Leuven, Belgium
^6^  Department of Cardiothoracic Surgery, Copenhagen University Hospital, Rigshospitalet, 2100 Copenhagen, Denmark
^7^  Division of Thoracic Surgery, Cardiac, Thoracic and Vascular Department, University Hospital of Pisa, 56124 Pisa, Italy
^8^  Department of Thoracic Surgery, University Hospitals Leuven, 3000 Leuven, Belgium
^9^  KU Leuven, Laboratory of Respiratory Diseases and Thoracic Surgery (BREATHE), CHROMETA, 3000 Leuven, Belgium
^10^  Department of Thoracic Surgery, Azienda Ospedaliera Universitaria Città della Salute e della Scienza di Torino, 10126 Torino, Italy
^11^  Department of Surgical Science, Section of Thoracic Surgery, University of Torino, 10126 Torino, Italy
^12^  Service of Thoracic Surgery, University Hospital of Lausanne, 1011 Lausanne, Switzerland
^13^  Department of Thoracic Surgery, Salamanca University Hospital, 37007 Salamanca, Spain
^14^  Unit of Thoracic Surgery, Fondazione Policlinico Universitario A. Gemelli IRCCS, 00168 Roma, Italy
^15^  Department of Thoracic Surgery, University Hospital Bordeaux, 33604 Pessac, France
^16^  Department of Cardiac and Thoracic Surgery, University Hospital Limoges, 87042 Limoges, France
^17^  Department of Thoracic Surgery, University Hospital Zürich, 8091 Zürich, Switzerland
^18^  Section of Thoracic Surgery, Department of Precision and Regenerative Medicine and Ionian Area, University of Bari “Aldo Moro”, 70124 Bari, Italy
^19^  Department of Thoracic Surgery, Lung Transplantation and Oesophageal Diseases, North Hospital, 13015 Marseille, France
^20^  Department of General and Cardiothoracic Surgery, University Hospital Rouen, 76031 Rouen, France
^21^  Department of Thoracic Surgery, Marmara University School of Medicine, 34854 Istanbul, Turkey
^22^  Department of Digestive Oncology, University Hospitals Leuven, 3000 Leuven, Belgium
^23^  Department of Thoracic Surgery, Bethanien Klinik, 8044 Zürich, Switzerland

*   Correspondence: elena.prisciandaro@outlook.com

With this correction, the order of the original affiliations 6 and 7 has been adjusted accordingly to affiliations 8 and 9. Meanwhile, the position of “KU Leuven” and the name of “CHROMETA” have been changed.

The corrected Author Contributions statement appears here.

**Author Contributions:** Conceptualization, E.P., L.B., S.F. and L.J.C.; methodology, E.P., L.B., S.F. and L.J.C.; software, E.P. and S.F.; validation, E.P. and L.B.; formal analysis, E.P., L.B., S.F. and L.J.C.; investigation, E.P., L.B., A.C., L.S., L.H., R.H.P., M.L., M.G.M., A.B., P.D.L., M.R., E.R., A.D., M.G., M.G.F.-G., C.F.-B., M.T.C., S.M., Y.B., M.T., J.T., P.F., N.L., I.O., A.D.P., G.M., C.B., P.A.T., F.M., J.-M.B., B.S., B.Y., J.D., D.E.V.R., W.W. and L.J.C.; data curation, E.P. and L.B.; writing—original draft preparation, E.P., L.B., S.F. and L.J.C.; writing—review and editing, E.P., L.B., S.F., A.C., L.S., L.H., R.H.P., M.L., M.G.M., A.B., P.D.L., M.R., E.R., A.D., M.G., M.G.F.-G., C.F.-B., M.T.C., S.M., Y.B., M.T., J.T., P.F., N.L., I.O., A.D.P., G.M., C.B., P.A.T., F.M., J.-M.B., B.S., B.Y., J.D., D.E.V.R., W.W. and L.J.C.; visualization, E.P. and L.B.; supervision, S.F. and L.J.C.; project administration, E.P.; funding acquisition, L.J.C. All authors have read and agreed to the published version of the manuscript.

The Acknowledgments section has been deleted accordingly.

The authors state that the scientific conclusions are unaffected. This correction was approved by the Academic Editor. The original publication has also been updated.
